# Successful Application of Alpha Lipoic Acid Niosomal Formulation in Cerebral Ischemic Reperfusion Injury in Rat Model

**DOI:** 10.34172/apb.2022.058

**Published:** 2021-09-12

**Authors:** Mohammad Amin Raeisi Estabragh, Abbas Pardakhty, Saeid Ahmadzadeh, Shahriar Dabiri, Reza Malekpour Afshar, Mohammad Farajli Abbasi

**Affiliations:** ^1^Pharmaceutics Research Center, Institute of Neuropharmacology, Kerman University of Medical Sciences, Kerman, Iran.; ^2^Student Research Committee, Kerman University of Medical Sciences, Kerman, Iran.; ^3^Pharmaceutical Sciences and Cosmetic Products Research Center, Kerman University of Medical Sciences, Kerman, Iran.; ^4^Pathology and Stem Cell Research Center, Afzalipour Faculty of Medicine, Kerman University of Medical Sciences, Kerman, Iran.; ^5^Neuroscience Research Center, Institute of Neuropharmacology, Kerman University of Medical Sciences, Kerman, Iran.

**Keywords:** Niosome, Alpha-lipoic acid, Cerebral ischemia, Antioxidant

## Abstract

***Purpose:*** Free radicals such as hydroxyl and peroxide are contributing factors to neuronal destruction in cerebral ischemia. Alpha-lipoic acid (ALA) is one of the potent known antioxidants. Preparation of ALA niosomes allows IV injection and can increase bioavailability and penetration into the central nervous system (CNS).

***Methods:*** Film hydration method was used to prepare different niosomes composed of Span®, Tween®, and cholesterol at different molar ratio. ALA and niosome-forming compounds were dissolved in chloroform, before removing the organic solvent by rotary evaporator. Animals were randomly divided into four groups: Sham, control group, intravenous (IV) injection of empty niosomes plus intraperitoneal (IP) injection of ALA solution, and finally, IV injection of ALA niosomes. Rats were subjected to deep anesthesia before inducing cerebral ischemia, then, their internal common carotid arteries were clamped for 15 min and reperfusion was done for 30 min. Niosomal ALA was injected intravenously just before declamping.

***Results:*** Mean volume diameter of the prepared niosomes was between 4.36 ± 0.82 and 19.95 ± 1.21 μm in different formulations. Encapsulation efficiency percent (EE%) of ALA in the selected formulation, Span60/Tween60/cholesterol (35:35:30 molar ratio), was 94.5 ± 0.2, and 59.27 ± 5.61% of ALA was released after 4h. In the niosomal group, the rate of reduction in complications of cerebral ischemia such as histopathologic changes and acute damage (from score 3 to 1) in CNS was higher than other groups.

***Conclusion:*** The obtained results show that niosomes can be used as effective drug delivery systems for ALA in cerebral ischemia.

## Introduction

Cerebral ischemia is one of the major causes of mortality worldwide. Cerebral ischemia reperfusion (I/R) injury is also one of the major causes of permanent physical and other irreversible disabilities.^1^ Eighty percent of strokes are related to the cerebral ischemia.^2,3^ Sixty percent of cerebral ischemia are thrombotic, and 40% are embolic.^4^ The only drug approved by the Food and Drug Administration (FDA) for use in acute ischemic stroke is Alteplase (t-PA; tissue plasminogen activator); however, as drug side effects, it can increase the risk of bleeding and some of the more dangerous complications.^2,3^ Therefore, many attempts have been made to protect brain tissue after cerebral I/R injury with fewer side effects.

One of the influential factors in neuronal destruction in cerebral I/R injury is the formation of free radicals such as hydroxyl and peroxide.^5^ Studies have shown that the use of antioxidants could reduce the complications of cerebral I/R injury.^6^ In animal studies, the use of antioxidants has been shown to reduce the damage of cerebral ischemia and, the results indicate a positive role of these agents in reducing I/R injury.^7^

Alpha-lipoic acid (ALA) is one of the lipophilic vitamin-like potent antioxidants,^8^ also known as thioctic acid.^9^ In 1951, ALA was first defined as a coenzyme in the Krebs cycle.^10^ ALA is made from octatonic acid under the influence of lipoic acid synthase. The ALA (R isomer) is found in low amounts in vegetables, tomato, red meat, liver, and animal’s kidney.^11^ In human studies, the oral form of ALA has been investigated. A study by Shay et al has reported no adverse effects of ALA in a dose of 4.2 g/d for two years.^12^ ALA is effective in studies of various diseases, including diabetic neuropathy, cardiovascular diseases, oxidative stress, and relative inflammatory diseases such as Alzheimer’s and dementia, multiple sclerosis, rheumatoid arthritis, and asthma.^13-16^ Its positive effects on weight loss has also been reported.^9^ In animal studies, the intraperitoneally used ALA and Etanercept for reducing cerebral I/R injury has been studied, and its positive effects on peripheral tumor necrosis factor alpha (TNF-α) and downregulation of microglial activation have been observed.^17^

The rate and extent of antioxidant’s access to the brain influence their effects on the central nervous system (CNS).^18^ Lipid-based vesicular systems can provide increased bioavailability, penetration into the CNS, and controlled therapeutic activity.^19^

The use of lipid-based vesicular systems for increasing the bioavailability of antioxidants, and their physicochemical stability has been studied.^20^ Niosomes, as an important class of lipid-based vesicular systems, with unique benefits, have attracted much attention as the high-potential drug delivery carriers over the past 35 years. Because of high stability, low cost of vesicle forming constituents, and easy formulation methods, niosomes are a good alternative for liposomes.^21^ Niosomes can be used for a variety of drugs and substances such as anti-Alzheimer’s, anti-cancer, antioxidant, and antibiotic drugs through various routes of administration such as oral, intravenous injections, and topical.^22,23^ Niosomes are considered as carriers that allow the intravenous administration of lipophilic molecules and their crossing through blood-brain barrier (BBB), which lead to increased CNS drug uptake.^24^ One mechanism proposed for better transport of niosomes through the BBB is their interaction with LDL receptors.^25^ Previous studies have shown increased penetration of niosomal formulations of drugs such as methotrexate and doxorubicin.^26,27^

There are several methods to prepare non-ionic surfactant vesicles (niosomes) such as thin film hydration (hand shaking), reverse-phase evaporation, ether or ethanol injection, dehydration rehydration, direct heating and sonication of lipids, and microfluidic hydrodynamic focusing method. Thin film hydration method is the most common procedure used for niosome preparation. In this method, all lipids and lipid soluble drugs are dissolved in an organic solvent, usually chloroform. Then, the solvent is evaporated under vacuum and lipid film is hydrated by aqueous phase containing components soluble in water at a temperature higher than the phase transition temperature of the used surfactants.^28^

In this study, ALA niosomes were studied based on their morphology, particle size analysis, encapsulation efficiency, physical stability, and *in vitro* ALA release. Finally, *in vivo* induction of I/R brain injury and the protection of ALA niosomes were also investigated on the basis of the great importance of niosomes properties on their performance in animal studies.^21^

## Materials and Methods

### Materials

ALA and cholesterol were purchased from Sigma-Aldrich Company (USA). The nonionic surfactants used as vesicle-forming materials containing sorbitan esters (Span^TM^ 20, 40, and 60) and polyoxyethylene sorbitan esters (Tween^TM^ 20, 40, and 60) were obtained from Fluka Company (Switzerland). All organic solvents and the other chemicals were of analytical grade and obtained from Merck Chemical Company (Germany).

### Preparation of ALA niosomes

Niosomal suspensions were formulated by the previously reported film hydration method.^29^ ALA niosomes were prepared using equimolar percent of the same surfactants hydrocarbon chain (C_12_, C_16_, and C_18_ Span/Tweens mixture) and different molar ratios (m.r.) of cholesterol according to Table 1. ALA, surfactants, and cholesterol were dissolved in chloroform; the organic solvent was removed in a rotary evaporator (Heidolph, Germany) at 60°C. Then, normal saline was added to the dried lipid film and rotated at 180 rpm and 60°C for 30 minutes. The formed lipid vesicles were stored at room temperature for 24 hours in borosilicate glass vials, and then, in a refrigerator for more studies. The final concentration of ALA in niosomes was 10 mg/mL.

**Table 1:** Composition of different niosomal formulation containing ALA

**Name**	**Constituents of lipid phase**	**Molar ratio**
F 1	Span20/Tween20/Cholesterol	25/25/50
F 2	Span20/Tween20/Cholesterol	30/30/40
F 3	Span20/Tween20/Cholesterol	35/35/30
F 4	Span40/Tween40/Cholesterol	25/25/50
F 5	Span40/Tween40/Cholesterol	30/30/40
F 6	Span40/Tween40/Cholesterol	35/35/30
F 7	Span60/Tween60/Cholesterol	25/25/50
F 8	Span60/Tween60/Cholesterol	30/30/40
F 9	Span60/Tween60/Cholesterol	35/35/30

### Characterization of ALA niosomes

#### Morphologic study of niosomes

The shape of niosomes as round and tubular ones, the type of vesicles as multilamellar vesicle (MLV), unilamellar vesicle (LUV), and small unilamellar vesicle (SUV), the aggregation of vesicles and separation of constituents’ particles were morphologically characterized by a light microscope (Leitz, HM-LUX3, Germany) equipped by a digital camera with 400X magnification.

### Size analysis

Mean volume diameters (d_V50_), vesicle size distribution and “span” of particles population were calculated by static laser light scattering (Malvern MasterSizer 2000E, UK) technique. The span that represents dispersity (from almost monodisperse to highly polydisperse) was calculated by Eq. (1):


(1) $$Span=\left(d_{V90}-d_{V10}\right)/d_{V50}$$

where *d*_V90_ and *d*_V10_ are cumulative 90 and 10% undersize volume size distribution, respectively.

### Physical stability of prepared formulations

To evaluate niosomal physical stability, formulations were kept at 4-8°C. Size analysis was done by statistic laser light scattering method at some defined time intervals including 1 week, 1 month, and 3 and 6 months after preparation of niosomes.

### Encapsulation efficiency percent (EE%)

Free ALA was separated from the encapsulated drug by dialysis method.^30^ Briefly, one mL of niosomal formulation in cellulose acetate membrane (Visking tube, MW cut off 12 KD) was exposed to 200 mL of water and ethanol (80:20 v/v%) during 4 hours at room temperature. Permeated free drug concentration was determined by the electrochemical method in the dialysate. The entrapped amount of ALA was also determined after adding one ml of isopropyl alcohol into niosomal suspension in dialysis bag content for disruption of niosomal bilayers.^31^ Encapsulation efficiency (EE%) was determined using Eq. (2):


(2) EE00=CencapCencap+CF×100

where *C*_encap_ and *C*_F_ denote the amount of ALA encapsulated in niosomes and free amount of ALA, respectively.

### Determination of ALA concentration

The method used for ALA determination was previously described.^32^ Briefly, by some modification, a three-electrode system including modified carbon paste electrode (MCPE), platinum wire, and Ag/AgCl (3 M KCl) employed as the working, counter, and reference electrodes, respectively, was connected to Autolab PGSTAT204-Metrohm potentiostat/galvanostat for electrochemical investigations. To fabricate the MCPE, a specific ratio of graphite powder and paraffin oil was poured into a mortar and mixed by grinding. Five percent w/w of ZnFe_2_O_4_ nanoparticles and 10.0% w/w of 1-butyl-3-methylimidazolium tetrafluoroborate as a conductive binder was added. The square-wave voltammetry (SWV) technique was employed for the quantitative determination of ALA concentration using the phosphate buffer solution of 0.1 M for pH adjustment at 6.3.

### In vitro release of ALA from niosomes

The release rate was evaluated using all-glass Franz diffusion cell at 37°C. Cellulose acetate dialysis tube (Visking tube, MW cut off 12 KD) was used as the membrane, after 24 hours hydration in recipient phase, water, and ethanol (80:20 volume %). The volume of the receptor compartment was 15 mL. Samples were taken at 0, 15, 30, 60, 120, 180, and 240 minutes near stirring magnet followed by replacing the fresh solvent in the receptor phase, and cumulative released percent was plotted against time.

Different kinetic models, including zero order, first order, Higuchi, Peppas, and Hixson-Crowell were assessed to determine the best kinetic model for releasing ALA niosomes.^33^

### In vivostudy

Twenty male Wistar rats (220-270 g) were used in this study. Animals were kept at a temperature of 20-22°C, 12/12 h dark/light cycle and had free access to food and water (*ad libitum*) before surgical procedures. Animals were anesthetized via intraperitoneal (IP) injection of diazepam (5 mg/kg) and ketamine (87 mg/kg) cocktail. To induce cerebral I/R injury, their internal common carotid arteries were clamped by Bulldog Clamp for 15 minutes, and then, reperfusion was established for 30 minutes.

Animals were randomly divided into four groups: Sham (group I), control group (group II), receiver of intravenous empty niosomes and intraperitoneal (IP) injection of ALA solution (group III), intravenous recipient of ALA niosomes (group IV), (n = 5). Animals in the sham surgery group underwent most of the surgical procedures involved but their internal common carotid arteries were not clamped as a control to eliminate the effect of pre-clamping surgery procedure on the brain injury.

Niosomes containing ALA (50 mg/kg) were injected intravenously immediately before releasing the clamp in group I. In group II, the equivalent volume of empty niosomes intravenously and the equivalent concentration (50 mg/kg) of ALA in ethanol and propylene glycol mixtures were injected IP.

### Histopathology

Animals were sacrificed by intravascular anesthetic agent high dose injection 30 min after reperfusion was performed. The brains were removed and fixed in 10% buffered formalin for at least 24 hours. The specimens were blocked and embedded in paraffin wax; 5 µm sections were obtained. The sections were stained with hematoxylin and eosin (H&E) for histopathologic scoring by light microscopy examination.

To semi-quantitatively assess the number of affected neurons (Table 2) in the hippocampus, the number of Eosinophilic neurons were counted under light microscopy by a veterinary pathologist who was blinded to the experimental groups. Those cells showing a distinct nucleus and nucleolus were defined as viable normal neurons.

Another histopathological assessment (Table 2) was also performed to investigate the quality of hippocampal neurons. The scales of this study allow the evaluation of I/R injuries that occur as a function of effect post-ischemia, as well as the rate of damage severity.

**Table 2:** Histopathological scoring of affected neurons numbers and hippocampus acute damage neurons

**Score**	**Numbers of affected neurons**	**Score of cerebral tissue acute damage**
0	Normal	Normal
1	Few affected neurons	Normal nucleus, scalloping at the cytoplasmic boarder
2	Many affected neurons	Nuclear shrinking, smeared nuclear-cytoplasmic detail
3	All neurons affected	Shrunken neuron with no nuclear-cytoplasmic detail

### Statistical analysis

Statistical significance was determined by Kruskal-Wallis test and Mann-Whitney test using SPSS software (SPSS for Windows, Version 20, IBM, SPSS Inc.). Statistical significant level was considered at *P* < 0.05.

## Results and Discussion

### Niosomes morphology

**Figure 1:**
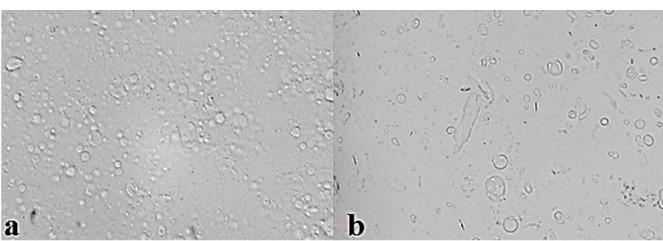
Optical micrographs of ALA niosomes (×400 magnification): (a) F9, (b) F1.

Span^®^ and Tween^®^ can be known as the most widely applied surfactants used for niosome preparation. The hydrophilic head groups of amphiphilic molecules make water-mediated interactions counter the previously formed force, finally result in bilayer formation. Cholesterol is known to abolish the gel to the liquid phase transition of niosomes, resulting in less leakiness of the vesicles and improved niosomes stability by forming hydrogen bonds between the hydroxyl group of cholesterol and the oxygen at the ester groups, or with the other functional oxygen groups of used surfactants.^34^ All used nonionic surfactant and cholesterol compositions at different molar ratios formed niosomes. The number of formed lipid vesicles was more remarkable in some Span/Tween 20 (ST20) niosomes (F1 and F3) and all Span/Tween 60 (ST60) formulations (F7, F8, and F9); therefore, these formulations were chosen for further studies as shown in Table 3. As shown in Figure 1, and also, on the basis of the size analysis results, most of lipid vesicles are as MLVs in the presence of cholesterol, which was predictable for film hydration method.

**Table 3:** Mean volume diameters (dv50%) and encapsulation Efficiency percent of ALA in selected niosomal formulations

**Formulation name**	**dv**_50%_ ** (µm) ± SD**				** Mean span (obtained by Eq. 1) **				**%EE (Mean ± SD; n=3)**
	**1 week**	**1 month**	**3 months**	**6 months**	**1 week**	**1 month**	**3 months**	**6 months**	
F 1	19.95±1.21	45.71±2.31	138.03±1.87	208.1±6.12	1.44	2.00	1.79	1.36	86.1±0.3
F 3	10.00±1.13	34.67±1.81	52.48±2.70	60.25±2.11	2.18	1.96	1.72	1.73	82.3±0.5
F 7	10.60±1.61	8.70±1.08	11.48±1.38	13.18±1.65	3.15	3.03	3.60	3.55	90.5±0.2
F 8	11.20±1.84	11.68±1.91	15.31±2.3	17.37±1.88	2.60	2.68	3.10	5.65	89.1±0.6
F 9	4.36±0.82	3.31±0.89	3.50±0.91	4.61±1.01	2.41	2.80	3.04	2.81	94.5±0.2

### Size analysis and physical stability of ALA niosomes

**Figure 2:**
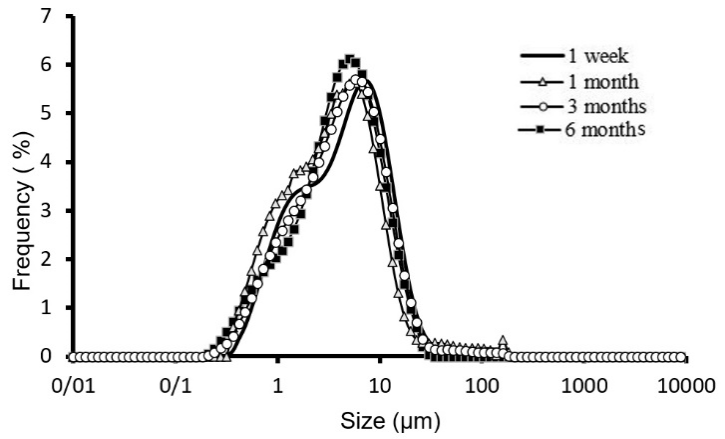
Physical stability of F 9 formulation kept at 4-8°C at 1 week, 1month, 3 and 6 months.

Mean volume diameters (dv_50%_) of the selected formulation of ALA niosomes prepared with different compositions are presented in Table 3 and the size distribution curve of formulation F9 of ALA niosomes is shown in Figure 2. Particle size distribution is one of the most essential characteristics in the examination of physical stability. The particle size distributions curve of the formulations were mostly Log-normal. In the formulation F9, this curve had the slightest change after six months, indicating appropriate physical stability of the formulation. The mean span of different prepared formulations is also presented in Table 3. The mean volume diameter of liquid state ST20 niosomes was larger than that of gel state ST60 ones (Table 3, *P* < 0.01). This can be attributed to higher hydrophilic-lipophilic balance (HLB) of ST20 compared to ST60 surfactants. This finding was also obtained in our previous research on insulin niosomes.^35^ Yoshioka et al^36^ explained this finding as a decrease in surface energy with increasing hydrophobicity (low HLB), resulting in the smaller vesicles at the same cholesterol ratio. Agarwal et al^37^ also reported that the mean size of primaquine phosphate-loaded niosomes showed a regular increase with increasing HLB from Span 85 (HLB 1.8) to Span 20 (HLB 8.6). Nowroozi et al^38^ obtained the same finding in the preparation of Brij 72, Tween 60, and Span 60 niosomes. On the other hand, it was shown that cholesterol had more effect on ordering surfactant molecules in niosomal bilayers of ST20 vesicles than ST60 one according to their span values of size distribution curves (Table 3).

Increasing cholesterol molar ratio from 30 to 50 led to larger niosomes in both ST20 and ST60 formulations (Table 3, *P* < 0.01). Based on the amphipathic nature of cholesterol molecules and reducing the curvature of lipid bilayers following cholesterol content increment, a more thermodynamically stable vesicle is established by forming larger niosomes.^39^ However, the most stable formulation was F9, which is composed of amphiphiles with C_18_ hydrocarbon chains and 30 cholesterols molar ratio content (Figure 2).

The span indicates the broad particle size distribution diagram and is expressed along with the dv_50%_ of any formulation. Increasing the span indicates an increase in the distance between the dv_10%_ and dv_90%_ toward dv_50%_. For any formulation at first, maybe particles with smaller diameters join together and decrease the difference between the dv_10%_ and dv_90%_, then, with the joining of medium-sized particles and the formation of larger particles, this number increases. F9 has more stable, lower diameter change and according to the dv_50%_ span is decreasing in 6 months for this formulation.

### Encapsulation efficiency

For quantitative determination of ALA, the SWV technique was used. This technique is known as a simple and efficient tool to quantify a large number of drugs, physiologically active substances, and other important chemicals. It also provides elegant methods to get access to relevant kinetic and thermodynamic parameters related to many lipophilic compounds and antioxidants such as ALA.

**Figure 3:**
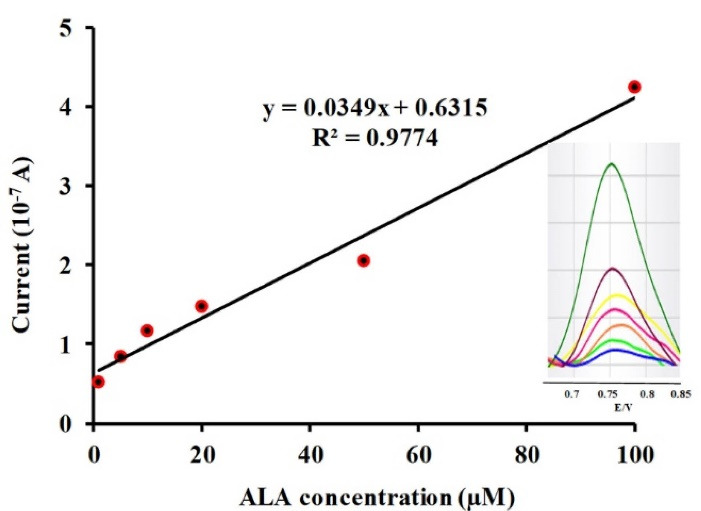
Plot of peak current as a function of ALA concentration. Inset shows the square wave voltammetry of modified carbon paste electrode in phosphate buffer solution (pH 6.3) correspond to different concentrations of ALA including: 1, 5, 10, 20, 50, 100 µM.

Figure 3 revealed an adequate linear response over the ALA concentration range of 1-100 µM versus the observed electro-oxidation current. The linear regression equation for the obtained calibration curve is expressed as below:


(3) $$I_{P}\left(10^{-7}A\right)=0.0349C_{ALA}+0.6315\left(R^{2}=0.9774\right)$$

After separating unentrapped ALA by dialysis method, concentration of drug encapsulated in the selected niosomal formulations was evaluated and the obtained EE% is presented in Table 3. Encapsulation and release of the drug from vesicular formulations such as niosomes and liposomes significantly depend on the physicochemical properties of the drug, bilayer formation amphiphiles, and cholesterol content. ALA is intercalated in lipid bilayers with high efficiency (higher than 82.3 ± 0.5%, Table 3) due to its lipophilic structure (LogP 2.75). Similar EE% was also reported by Zhao et al^40^ in chitosan-coated liposomes. The EE% of ALA in the niosomal formulations of ST20 and ST60 decreased with increasing cholesterol content, which may be due to the competition of cholesterol and hydrophobic compounds for incorporation into the bilayers of lipid vesicles.^41^ In general, as the hydrocarbon chain length of used surfactants increases, the EE% of lipid soluble drugs such as carvedilol^29^ and flurbiprofen^42^ is increased. In this study, ST60 niosomes with C_18_ alkyl chain also depicted more EE% compared to ST20 with C_12_ hydrocarbon chain (Table 3).

### In vitro release studies

**Figure 4:**
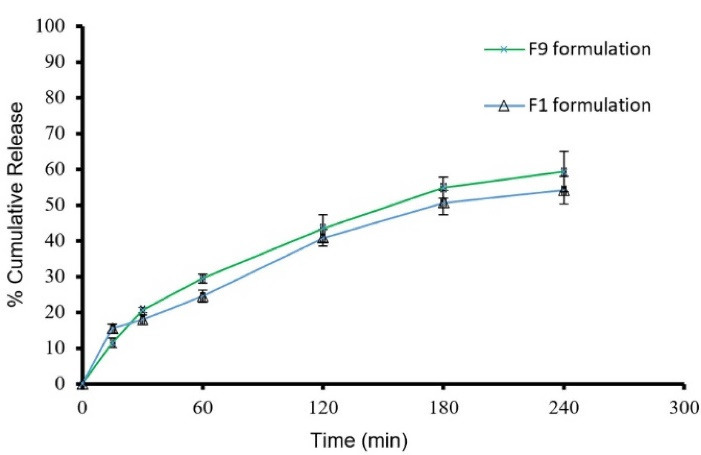
Cumulative release percent in F9 and F1formulations (mean ± SD, n = 3)

Based on morphology, size distribution, and drug encapsulation efficiency of the prepared niosomes, formulations F1 and F9 were selected for release of ALA; the results are shown in Figure 4. After 4 hours, F1 and F9 have 54.13 ± 3.91 and 59.27 ± 5.61% release of drug content, respectively. The release kinetic data of Formulation F9 were fitted to different models by the regression coefficient of determination (R^2^). Parameters values of relevant kinetics models are presented in Table 4. The best model fitted with release data was Higuchi (R^2^ = 0.9914 and K = 3.9128), which showed the prominent diffusion mechanism of ALA through lipid bilayers.^43^ To confirm diffusion mechanism, the data were fitted into Korsmeyer-Peppas model, and non-Fickian release explained the main mechanism of ALA delivery according to *n* value (0.60, 0.45 < n < 0.85).

**Table 4:** Histopathological evaluation of affected neuron numbers subsequent to cerebral I/R injury.

**Group**	**Score of affected neuron numbers**	**Score of cerebral tissue acute damage**
I	0 (0)	0 (0)
II	2 (2-3)^a^	2 (2-3)^a^
III	2 (2-3)^a^	3 (2-3)^a^
IV	1 (0-1)^ab^	1 (1)^ab^

(a) and (b) letters show significant difference (*P* < 0.05) in comparison with sham and control groups, respectively. Scores in parentheses indicate the distribution of animal scores in each group.

Incomplete delivery of ALA from niosomes could be attributed to reducing release driving force, concentration gradient, in Franz diffusion cell.^44^ This pattern of release from niosomes was reported for tioxolne,^45^ naltrexone HCl,^46^ and caffeine.^47^ Furthermore, chemical interactions such as hydrogen bonding could be one of the incomplete or low release of encapsulated compound.^48^

In vitro release data are often used for delivery ranking of encapsulated compound, and also, for predicting the release kinetic model or fitting with existing models.^49^ This discrepancy is due to the large biologic membrane pool present in human body cells, into which drugs can distribute after in vivo administration. However, in some studies, this method could be used *in vitro* correlation release data and *in vivo* behavior of the used formulation.^50^

### Animal studies

**Figure 5:**
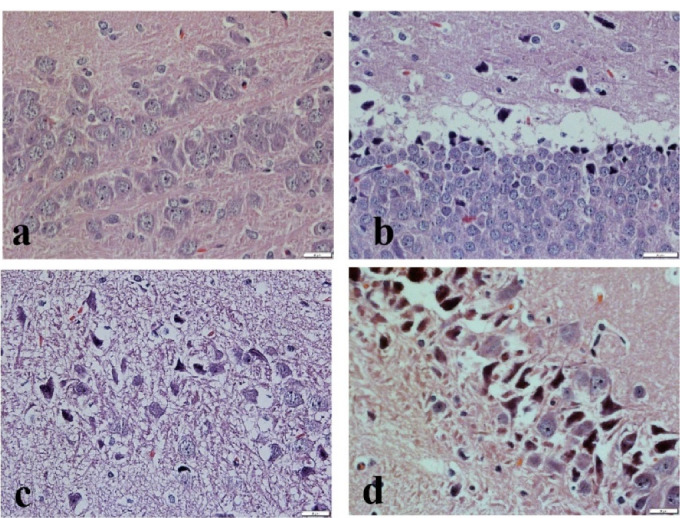
The rat’s hippocampus sections stained with hematoxylin and eosin. (a) Normal nucleus neural cells; (b) Normal nucleus with scalloping at the cytoplasmic boarder; (c) Nuclear shrinking neural cell, along with smeared nuclear-cytoplasmic detail; (d) Shrunken neuron with no nuclear-cytoplasmic detail.

Histopathological scores taken after cerebral I/R injury are reported in Table 4. The hippocampus sections of group I revealed the normal neural cell structure without any signs of cytoplasmic scalloping or shrinking (Figure 5a). The histopathologic scores of the affected neuron number and acute damage in this group were considered zero (Table 4).

The shrunken neurons with the disappearance of cytoplasmic detail related to cerebral I/R injury were seen extensively in groups II and III. These animals exhibited macrophages and astrocytes around necrotic hippocampal regions. Relative to group I, groups II and III had significantly fewer viable cells and most of them were affected by I/R injury at hippocampus sections (Figure 5c, d) (*P* < 0.05). Tissue inflammation and degenerative changes were cleared in all tissue samples of groups (II, II, IV) with different levels. The animals in group IV showed neuronal loss by I/R injury cytoplasmic eosinophilia and nuclei pinkies, but it had a significant difference with groups II and III in viable cell count and damage amount (Table 4). It could be related to treatment used in the present study.

The complication of cerebral ischemia is one of the main reasons that leads to mortality, permanent and irreversible disability (1). Various factors such as primary cerebrovascular embolism or subsequent brain injury are the main causes of ischemia in the brain.^51^ However, intertwined pathophysiological processes such as apoptosis, necrosis, and autophagy due to the release of free radicals from ischemic area following reperfusion are the main factors of tissue damage in I/R injury.^5,52^

In the Lapchak study, the use of antioxidants reduced the complications of cerebral ischemia.^6^ For example, de Sales et al,^53^ used ascorbic acid (vitamin C) as an antioxidant in an animal model of cerebral I/R injury and the obtained results showed the neuroprotective effect of it. However, vitamin C as a water soluble antioxidant has a low capability to pass through BBB. On the other hand, lipid soluble antioxidants such as ALA cannot be IV injected to provide a high concentration in damaged brain tissue. Lipid vesicles such as niosomes can be used as targeted drug delivery systems to CNS, due to the enhanced penetration effect into the brain tissue.^24,54^ Varshosaz et al^7^ showed the benefits of niosomal formulation of vitamins C and E in reducing the adverse effects of brain ischemia in an animal model.

ALA is considered as an ideal antioxidant due to its unique properties such as rapid absorption, antioxidant effects in both its reduced and oxidized forms, and its ability to chelate free metal ions.^10,55^ The positive effects of ALA in the treatment or reduction of injuries caused by various disorders such as neurovascular, cardiovascular, metabolic, urogenital, and ischemic injuries in various organs have been proven in previous studies.^56^ For example, ALA inhibits oxidative stress‐induced apoptosis in brain injury with possible involvement of the Nrf2 pathway.^57^ ALA also reduces the effects of ischemia on cardiomyocytes by reducing the autophagy of heart tissue cells.^52^ In the study of Wu et al,^17^ ALA and Etanercept were used to protect cerebral I/R injury. Turkyilmaz et al^58^ reported that treatment with ALA (50 mg/kg/d) can reverse some adverse effects of valproic acid such as reducing total antioxidant capacity by decreasing glutathione, superoxide dismutase, catalase and increasing lipid peroxidation led to brain injury. In acute phase, ALA therapy can protect the brain against I/R injury and improve neurological functions with modulating mTOR signaling pathway.^56^ Also, ALA and *Ginkgo biloba* signiﬁcantly reduced pro-inﬂammatory mediators such as TNF-α and IL-1β, and improved the serious harmful effects of silver nanoparticles on BBB and neurons function. ALA showed a novel therapy to stabilize the integrity of BBB, due to its antioxidant, anti-inflammatory, and anti-apoptotic functions.^59^ Capability of surfactants to form bilayers and the presence of cholesterol, as the main sterol in biological membranes, in niosomal structure leads to the use of this type of lipid vesicles for transport of different materials such as pramipexole^60^ and rivastigmine^61^ through BBB. The results of the present study also showed that the prepared niosomal formulation of ALA capable it to be intravenously administered and increase the pharmacological effect of ALA compared to other group that receive free ALA. IV administration of ALA niosomes (50 mg/kg) diminished shrunken neurons and significantly increased viable cells compared to the other groups in the rat model of cerebral I/R injury (Table 4 and Figure 5c, d) (*P* < 0.05). Based on the cerebrospinal fluid osmolarity levels, a similar result was observed when ALA therapy successfully reduced brain edema after traumatic injury.^10^

## Conclusion

According to the results of this study, utilized Span/Tween/cholesterol mixtures could form stable ALA niosomes and the selected formulation (Span60/Tween60/Cholesterol, 35:35:30 m.r.) was successfully used for treatment of cerebral I/R injury in the rat model. The results were promising for further *In Vivo* applications of ALA niosomes in brain injuries and neurodegenerative diseases induced by oxidative stress and free radicals production.

## Acknowledgments

This study was approved and financially supported by Kerman Neuroscience Research Center (No.97-22). Also, the authors would like to thank the Vice Chancellor for Research and Technology of Kerman University of Medical Sciences (KMU) for supporting this study (Proposal Number: 9700648).

## Ethics Issues

All animal study procedures were approved by the Biomedical Ethics Committee of Kerman University of Medical Sciences (Ethical code: IR.KMU.REC.1398.445).

## Conflict of Interest

Authors declare no conflict of interest in this study.

## References

[1] Katan M, Luft A (2018). Global Burden of Stroke. Semin Neurol.

[2] Kernan WN, Ovbiagele B, Black HR, Bravata DM, Chimowitz MI, Ezekowitz MD (2014). Guidelines for the prevention of stroke in patients with stroke and transient ischemic attack: a guideline for healthcare professionals from the American Heart Association/American Stroke Association. Stroke.

[3] Powers WJ, Rabinstein AA, Ackerson T, Adeoye OM, Bambakidis NC, Becker K (2018). 2018 guidelines for the early management of patients with acute ischemic stroke: a guideline for healthcare professionals from the American Heart Association/American Stroke Association. Stroke.

[4] Cassella CR, Jagoda A (2017). Ischemic stroke: advances in diagnosis and management. Emerg Med Clin North Am.

[5] Sinha J, Das N, Basu MK (2001). Liposomal antioxidants in combating ischemia-reperfusion injury in rat brain. Biomed Pharmacother.

[6] Lapchak PA (2010). A critical assessment of edaravone acute ischemic stroke efficacy trials: is edaravone an effective neuroprotective therapy?. Expert OpinPharmacother.

[7] Varshosaz J, Taymouri S, Pardakhty A, Asadi-Shekaari M, Babaee A (2014). Niosomes of ascorbic acid and α-tocopherol in the cerebral ischemia-reperfusion model in male rats. Biomed Res Int.

[8] Sampaio LRL, Cysne Filho FMS, de Almeida JC, dos Santos Diniz, Patrocínio CFV, de Sousa CNS (2018). Advantages of the alpha-lipoic acid association with chlorpromazine in a model of schizophrenia induced by ketamine in rats: behavioral and oxidative stress evidences. Neuroscience.

[9] Huerta AE, Fernández-Galilea M, Prieto-Hontoria PL, Martínez JA, Moreno-Aliaga MJ. Alpha-lipoic acid: a dietary supplement with therapeutic potential for obesity and related metabolic diseases. In: Nabavi SM, Silva AS, eds. Nonvitamin and Nonmineral Nutritional Supplements. Academic Press; 2019. p. 85-92. 10.1016/B978-0-12-812491-8.00011-4

[10] Kul H, Celik H, Kurtulus A, Tekiner A, Erdem Y, Kul G (2020). The effect of alpha lipoic acid on cerebrospinal fluid biochemistry and brain edema after experimental traumatic brain injury. Turk Neurosurg.

[11] Akiba S, Matsugo S, Packer L, Konishi T (1998). Assay of protein-bound lipoic acid in tissues by a new enzymatic method. Anal Biochem.

[12] Shay KP, Moreau RF, Smith EJ, Smith AR, Hagen TM (2009). Alpha-lipoic acid as a dietary supplement: molecular mechanisms and therapeutic potential. BiochimBiophys Acta.

[13] Moura FA, de Andrade KQ, dos Santos JC, Goulart MO (2015). Lipoic acid: its antioxidant and anti-inflammatory role and clinical applications. Curr Top Med Chem.

[14] Salinthone S, Yadav V, Bourdette DN, Carr DW (2008). Lipoic acid: a novel therapeutic approach for multiple sclerosis and other chronic inflammatory diseases of the CNS. EndocrMetab Immune Disord Drug Targets.

[15] Holmquist L, Stuchbury G, Berbaum K, Muscat S, Young S, Hager K (2007). Lipoic acid as a novel treatment for Alzheimer’s disease and related dementias. PharmacolTher.

[16] Zhao RR, Xu F, Xu XC, Tan GJ, Liu LM, Wu N (2015). Effects of alpha-lipoic acid on spatial learning and memory, oxidative stress, and central cholinergic system in a rat model of vascular dementia. Neurosci Lett.

[17] Wu MH, Huang CC, Chio CC, Tsai KJ, Chang CP, Lin NK (2016). Inhibition of peripheral TNF-α and downregulation of microglial activation by alpha-lipoic acid and etanercept protect rat brain against ischemic stroke. Mol Neurobiol.

[18] Abbott NJ, Romero IA (1996). Transporting therapeutics across the blood-brain barrier. Mol Med Today.

[19] Pardakhty A, Moazeni E (2013). Nano-niosomes in drug, vaccine and gene delivery: a rapid overview. Nanomed J.

[20] Junyaprasert VB, Singhsa P, Suksiriworapong J, Chantasart D (2012). Physicochemical properties and skin permeation of Span 60/Tween 60 niosomes of ellagic acid. Int J Pharm.

[21] Ge X, Wei M, He S, Yuan WE (2019). Advances of non-ionic surfactant vesicles (niosomes) and their application in drug delivery. Pharmaceutics.

[22] Puras G, Mashal M, Zárate J, Agirre M, Ojeda E, Grijalvo S (2014). A novel cationic niosome formulation for gene delivery to the retina. J Control Release.

[23] Muzzalupo R, Mazzotta E (2019). Do niosomes have a place in the field of drug delivery?. Expert Opin Drug Deliv.

[24] Gharbavi M, Amani J, Kheiri-Manjili H, Danafar H, Sharafi A (2018). Niosome: a promising nanocarrier for natural drug delivery through blood-brain barrier. Adv Pharmacol Sci.

[25] Ingallina C, Rinaldi F, Bogni A, Ponti J, Passeri D, Reggente M (2016). Niosomal approach to brain delivery: development, characterization and in vitro toxicological studies. Int J Pharm.

[26] Bragagni M, Mennini N, Ghelardini C, Mura P (2012). Development and characterization of niosomal formulations of doxorubicin aimed at brain targeting. J Pharm Pharm Sci.

[27] Azmin MN, Florence AT, Handjani-Vila RM, Stuart JF, Vanlerberghe G, Whittaker JS (1985). The effect of non-ionic surfactant vesicle (niosome) entrapment on the absorption and distribution of methotrexate in mice. J Pharm Pharmacol.

[28] Pardakhty A. Non-ionic surfactant vesicles (niosomes) as new drug delivery systems. In: Information Reso Management Association, ed. Pharmaceutical Sciences: Breakthroughs in Research and Practice. Hershey, PA: IGI Global; 2017. p. 154-84.

[29] Taymouri S, Varshosaz J (2016). Effect of different types of surfactants on the physical properties and stability of carvedilol nano-niosomes. Adv Biomed Res.

[30] Obeid MA, Khadra I, Albaloushi A, Mullin M, Alyamani H, Ferro VA (2019). Microfluidic manufacturing of different niosomes nanoparticles for curcumin encapsulation: physical characteristics, encapsulation efficacy, and drug release. Beilstein J Nanotechnol.

[31] Rameshk M, Sharififar F, Mehrabani M, Pardakhty A, Farsinejad A, Mehrabani M (2018). Proliferation and in vitro wound healing effects of the microniosomes containing Narcissus tazetta L. bulb extract on primary human fibroblasts (HDFs). Daru.

[32] Ferreira APM, dos Santos Pereira LN, da Silva IS, Tanaka SM, Tanaka AA, Angnes L (2014). Determination of α-lipoic acid on a pyrolytic graphite electrode modified with cobalt phthalocyanine. Electroanalysis.

[33] Shoaib MH, Al Sabah Siddiqi S, Yousuf RI, Zaheer K, Hanif M, Rehana S (2010). Development and evaluation of hydrophilic colloid matrix of famotidine tablets. AAPS PharmSciTech.

[34] Nematollahi MH, Pardakhty A, Torkzadeh-Mahanai M, Mehrabani M, Asadikaram G (2017). Changes in physical and chemical properties of niosome membrane induced by cholesterol: a promising approach for niosome bilayer intervention. RSC Adv.

[35] Varshosaz J, Pardakhty A, Hajhashemi VI, Najafabadi AR (2003). Development and physical characterization of sorbitan monoester niosomes for insulin oral delivery. Drug Deliv.

[36] Yoshioka T, Sternberg B, Florence AT (1994). Preparation and properties of vesicles (niosomes) of sorbitan monoesters (Span 20, 40, 60 and 80) and a sorbitan triester (Span 85). Int J Pharm.

[37] Agarwal S, Bakshi V, Vitta P, Raghuram AP, Udupa N (2004). Effect of cholesterol content and surfactant HLB on vesicle properties of niosomes. Indian J Pharm Sci.

[38] Nowroozi F, Almasi A, Javidi J, Haeri A, Dadashzadeh S (2018). Effect of surfactant type, cholesterol content and various downsizing methods on the particle size of niosomes. Iran J Pharm Res.

[39] Essa EA (2014). Effect of formulation and processing variables on the particle size of sorbitan monopalmitate niosomes. Asian J Pharm.

[40] Zhao GD, Sun R, Ni SL, Xia Q (2015). Development and characterisation of a novel chitosan-coated antioxidant liposome containing both coenzyme Q10 and alpha-lipoic acid. J Microencapsul.

[41] Socaciu C, Jessel R, Diehl HA (2000). Competitive carotenoid and cholesterol incorporation into liposomes: effects on membrane phase transition, fluidity, polarity and anisotropy. Chem Phys Lipids.

[42] Mokhtar M, Sammour OA, Hammad MA, Megrab NA (2008). Effect of some formulation parameters on flurbiprofen encapsulation and release rates of niosomes prepared from proniosomes. Int J Pharm.

[43] Mahdavi Moghddam SR, Ahad A, Aqil M, Imam SS, Sultana Y (2016). Formulation and optimization of niosomes for topical diacerein delivery using 3-factor, 3-level Box-Behnken design for the management of psoriasis. Mater Sci Eng C Mater Biol Appl.

[44] Shariat S, Hakimzadeh V, Pardakhty A (2020). The physicochemical and organoleptic evaluation of the nano/micro encapsulation of omega-3 fatty acids in lipid vesicular systems. Nanomed J.

[45] Hakimi Parizi M, Sharifi I, Farajzadeh S, Pardakhty A, Parizi M, Sharifi H (2019). Tioxolone niosomes exert antileishmanial effects on Leishmania tropica by promoting promastigote apoptosis and immunomodulation. Asian Pac J Trop Med.

[46] Abdelkader H, Ismail S, Kamal A, Alany RG (2011). Design and evaluation of controlled-release niosomes and discomes for naltrexone hydrochloride ocular delivery. J Pharm Sci.

[47] Khazaeli P, Pardakhty A, Shoorabi H (2007). Caffeine-loaded niosomes: characterization and in vitro release studies. Drug Deliv.

[48] Hao YM, Li K (2011). Entrapment and release difference resulting from hydrogen bonding interactions in niosome. Int J Pharm.

[49] Jain A, Jain SK. In vitro release kinetics model fitting of liposomes: an insight. Chem Phys Lipids 2016. 10.1016/j.chemphyslip.2016.10.005 27983957

[50] Shabbits JA, Chiu GN, Mayer LD (2002). Development of an in vitro drug release assay that accurately predicts in vivo drug retention for liposome-based delivery systems. J Control Release.

[51] Eltzschig HK, Eckle T (2011). Ischemia and reperfusion--from mechanism to translation. Nat Med.

[52] Zhang X, Yan H, Yuan Y, Gao J, Shen Z, Cheng Y (2013). Cerebral ischemia-reperfusion-induced autophagy protects against neuronal injury by mitochondrial clearance. Autophagy.

[53] de Sales KP, Pinto BA, Ribeiro NL, Melo TM, Galvão-Moreira LV, de Brito Filho SB (2019). Effects of vitamin C on the prevention of ischemia-reperfusion brain injury: experimental study in rats. Int J Vasc Med.

[54] Maestrelli F, Landucci E, De Luca E, Nerli G, Bergonzi MC, Piazzini V (2019). Niosomal formulation of a lipoyl-carnosine derivative targeting TRPA1 channels in brain. Pharmaceutics.

[55] Skibska B, Józefowicz-Okonkwo G, Goraca A (2006). Protective effects of early administration of alpha-lipoic acid against lipopolysaccharide-induced plasma lipid peroxidation. Pharmacol Rep.

[56] Gao X, Chen W, Li J, Shen C, Zhou P, Che X (2018). The protective effect of alpha-lipoic acid against brain ischemia and reperfusion injury via mTOR signaling pathway in rats. Neurosci Lett.

[57] Xia D, Zhai X, Wang H, Chen Z, Fu C, Zhu M (2019). Alpha lipoic acid inhibits oxidative stress-induced apoptosis by modulating of Nrf2 signalling pathway after traumatic brain injury. J Cell Mol Med.

[58] Turkyilmaz IB, Bilgin Sokmen B, Yanardag R (2020). Alpha-lipoic acid prevents brain injury in rats administered with valproic acid. J Biochem Mol Toxicol.

[59] Lebda MA, Sadek KM, Tohamy HG, Abouzed TK, Shukry M, Umezawa M (2018). Potential role of α-lipoic acid and Ginkgo biloba against silver nanoparticles-induced neuronal apoptosis and blood-brain barrier impairments in rats. Life Sci.

[60] Waddad AY, Abbad S, Yu F, Munyendo WL, Wang J, Lv H (2013). Formulation, characterization and pharmacokinetics of Morin hydrate niosomes prepared from various non-ionic surfactants. Int J Pharm.

[61] Kulkarni P, Rawtani D, Barot T (2021). Design, development and in-vitro/in-vivo evaluation of intranasally delivered Rivastigmine and N-Acetyl Cysteine loaded bifunctional niosomes for applications in combinative treatment of Alzheimer’s disease. Eur J Pharm Biopharm.

